# Bowel Perforation in the Emergency Department Related to Bevacizumab Therapy and Recurrent Ovarian Cancer

**DOI:** 10.5811/cpcem.2020.1.45374

**Published:** 2020-03-27

**Authors:** Stuart A. Ostby, Michael Olushoga, Charles A. Leath, Samuel L. Burleson

**Affiliations:** * University of Alabama at Birmingham, Department of Obstetrics and Gynecology, Birmingham, Alabama; † University of Alabama at Birmingham, Department of Emergency Medicine, Birmingham, Alabama; ‡ University of Alabama at Birmingham, Department of Obstetrics and Gynecology, Division of Gynecologic Oncology, Birmingham, Alabama

**Keywords:** bevacizumab, oncologic emergency, bowel ischemia

## Abstract

**Case Presentation:**

We describe the presentation to the emergency department of a patient with recurrent ovarian cancer treated with bevacizumab with the complication of bowel perforation.

**Discussion:**

We review the frequency and outcomes of bevacizumab-related bowel perforation. We also report the patient’s imaging findings, including the radiologic presentation of free intraperitoneal air and portal venous gas, both indicative of bowel perforation and the need for emergent surgical evaluation. Our case also illustrates the potentially catastrophic side effects of bevacizumab and other targeted oncologic therapies of which emergecny physicians may not be aware.

## CASE PRESENTATION

**Image 1:**
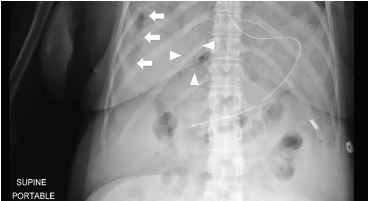
Upright abdominal radiograph revealing free air along the lateral margin of the liver (arrows) and branching gas in the liver (triangles), concerning for portal venous gas.

**Image 2:**
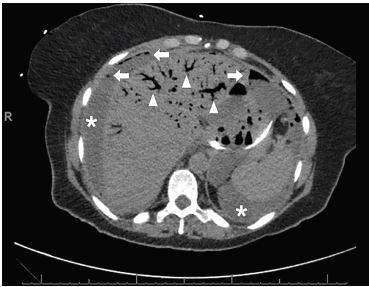
Computed tomography scan of the abdomen revealing extensive ascites (*), intraperitoneal free air (arrows), and extensive branching portal venous gas (triangles), indicative of bowel ischemia, necrosis, and perforation.

**Image 3:**
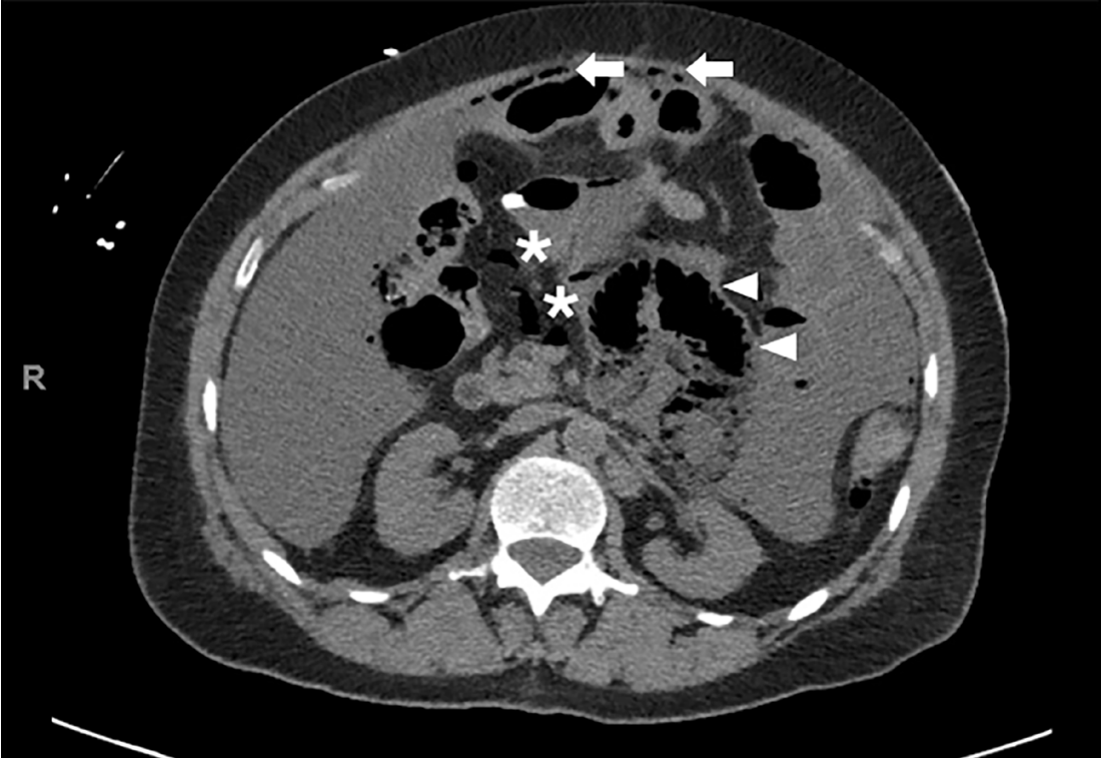
Computed tomography of the abdomen revealing intraperitoneal free air (arrows), bowel wall pneumatosis (triangles), and air in the mesenteric vasculature (*).

A 69-year-old, African-American female with recurrent stage IIIC ovarian carcinoma treated with bevacizumab presented to the emergency department (ED) with abdominal pain, distention, vomiting, and hypotension. After initial stabilization, an upright abdominal radiograph (Image 1) revealed peritoneal free air and portal venous gas concerning for bowel perforation, which was confirmed by computed tomography (CT) of the abdomen and pelvis (Images 2 and 3).

The patient was admitted to the gynecology oncology service and maintained on crystalloids and antibiotics. She had minimal symptoms. In accordance with her wishes, no further aggressive intervention was pursued, and she died on hospital day three.

## DISCUSSION

Bevacizumab is a monoclonal antibody targeting the vascular endothelial growth factor receptor used in multiple cancer types, including ovarian.^1^, ^2^ Complications include bowel perforation and gastrointestinal (GI) bleeding.^3^ The incidence of bowel perforation in ovarian cancer treated with bevacizumab is estimated to be 2–3%,^4^ with a relative risk of 2.57 compared to ovarian cancer alone.^5^ Bowel perforation and other severe GI pathologies are seen with other commonly-used targeted therapies such as sunitinib, sorafenib, everolimus, and temsirolimus.^6^ This patient had other independent risk factors for perforation including bowel resection-reanastamosis, peritoneal carcinomatosis, and partial small bowel obstructions.

**CPC-EM Capsule**

What do we already know about this clinical entity?

Bevacizumab is an increasingly used targeted chemotherapeutic agent with infrequent, severe complications including gastrointestinal perforation.

What is the major impact of the image(s)?

Severe ischemic bowel related to bevacizumab therapy and widespread diagnostic findings of mesenteric ischemia, portal venous gas, and free air are demonstrated.

How might this improve emergency medicine practice?

Early recognition and diagnosis for bowel perforation in patients on bevacizumab is essential to allow prompt surgical evaluation and therapy.

Bowel perforation secondary to bevacizumab has an estimated 60-day mortality of 25%.^2^ The diagnosis is confirmed by radiographs or CT of the abdomen demonstrating intraperitoneal free air, pneumatosis intestinalis, or portal venous gas. While management is generally surgical, supportive care, including antibiotics, parenteral nutrition, and fluid resuscitation, has been successful in poor surgical candidates.

In summary, we report a case of bowel perforation related to bevacizumab therapy. This case demonstrates the potentially life-threatening side effects of bevacizumab and other frequently-used, targeted therapies requiring ED diagnosis and resuscitation, and the complex imaging findings associated with the diagnosis of bowel perforation in a patient with recurrent ovarian cancer.
